# A case of the lower gastrointestinal bleeding due to Dieulafoy’s ulcer in the cecum

**DOI:** 10.1007/s12328-020-01109-4

**Published:** 2020-03-03

**Authors:** Keisuke Kinoshita, Osamu Matsunari, Akira Sonoda, Kensuke Fukuda, Kazuhisa Okamoto, Ryo Ogawa, Kazuhiro Mizukami, Tadayoshi Okimoto, Masaaki Kodama, Kazunari Murakami

**Affiliations:** 1 Department of Gastroenterology, Usa Takada Medical Association Hospital, 635 Minamiusa, Usa, Oita 872-0102 Japan; 2 Department of Gastroenterology, Oita University, 1-1 Idaigaoka, Hasama, Yufu, Oita 879-5593 Japan

**Keywords:** Dieulafoy’s ulcer, Cecum, Endoscopic clips

## Abstract

Gastrointestinal Dieulafoy’s ulcer is a rare disease of unknown etiology. Dieulafoy’s ulcer often presents in the stomach and is thought to cause about 5% of all gastrointestinal bleeds in adults, but can be found in any part of the gastrointestinal tract. Dieulafoy’s ulcer corresponds to an arterial malformation in the submucosal space and can cause life-threatening hemorrhage. We report a case of the lower gastrointestinal bleeding from a cecal Dieulafoy’s ulcer that was successfully treated with endoscopic clips. An 82-year-old woman had been diagnosed with hypertension and cerebral infarction. She had been using aspirin to prevent recurrent infarction. She was admitted to our hospital with hematochezia. Urgent colonoscopy revealed a small, reddish vascular malformation in the cecum. The lesion was suggestive of Dieulafoy’s ulcer and was treated with endoscopic clips. The patient has since been discharged from our hospital without experiencing any further bleeding. Endoscopy is a useful method for diagnosing and treating Dieulafoy’s ulcer.

## Introduction

Dieulafoy’s ulcer is a rare disease of unknown etiology, corresponding to a submucosal arterial malformation. Dieulafoy’s ulcer is said to often affect the elderly and men, with a male:female ratio of 2:1, and greater frequency in patients using non-steroidal anti-inflammatory drugs (NSAIDs), aspirin, or warfarin and in patients with hypertension, chronic kidney disease, or liver cirrhosis [1–3]. Effects can include fatal massive bleeding or recurrent bleeding from the same source. As a result, misdiagnosis and delayed treatment can lead to death. The most common location of Dieulafoy’s ulcer is the stomach, although any part of the gastrointestinal tract can be affected. Localization of the source of bleeding is usually achieved endoscopically, but diagnosis is very difficult, particularly for lesion located in the large intestine. We report a case of the lower gastrointestinal bleeding from a cecal Dieulafoy’s ulcer successfully treated with endoscopic clipping.

## Case report

An 82-year-old woman was admitted to our hospital with severe hematochezia. She had been diagnosed with hypertension and cerebral infarction and was using aspirin to prevent recurrent infarction. Consciousness was clear and vital signs were stable. Rectal examination did not reveal bright red blood, and no mass was palpable. Laboratory data revealed: white blood cell count, 3000/μl; red blood cell count, 263 × 10^4^/μl; hemoglobin, 7.8 g/dl; and platelet count, 10.5 × 10^4^/μl. Serum albumin level was 4.0 g/dl. Blood urea nitrogen level was 19.8 mg/dl. Creatinine level was 0.76 mg/dl. Levels of lactate dehydrogenase, liver enzymes, amylase, and electrolytes were normal (Table 1). She was treated with fluid resuscitation and blood transfusion.

**Table 1:** Laboratory data on admission

WBC	3000/μl	TP	6.4 g/dl	γ-GTP	36 U/l
RBC	263 × 10^4^/μl	Alb	4.0 g/dl	Amy	91 U/l
Hb	7.8 g/dl	BUN	19.8 mg/dl	Na	141 mEq/l
Hct	24.3%	Cre	0.76 mg/dl	Cl	108 mEq/l
MCV	92.4 fl	T-bil	0.4 mg/dl	K	4.4 mEq/l
MCHC	32.1%	CK	33 U/l	CRP	0.04 mg/dl
Plt	10.5 × 10^4^/μl	AST	17 U/l	HbA1c	5.9%
Ret	3.5%	ALT	11 U/l	HBs Ag	(–)
PT (%)	110%	LDH	117 U/l	HCV Ab	(–)
APTT	23.5 s	ALP	214 U/l		

**Fig. 1:**
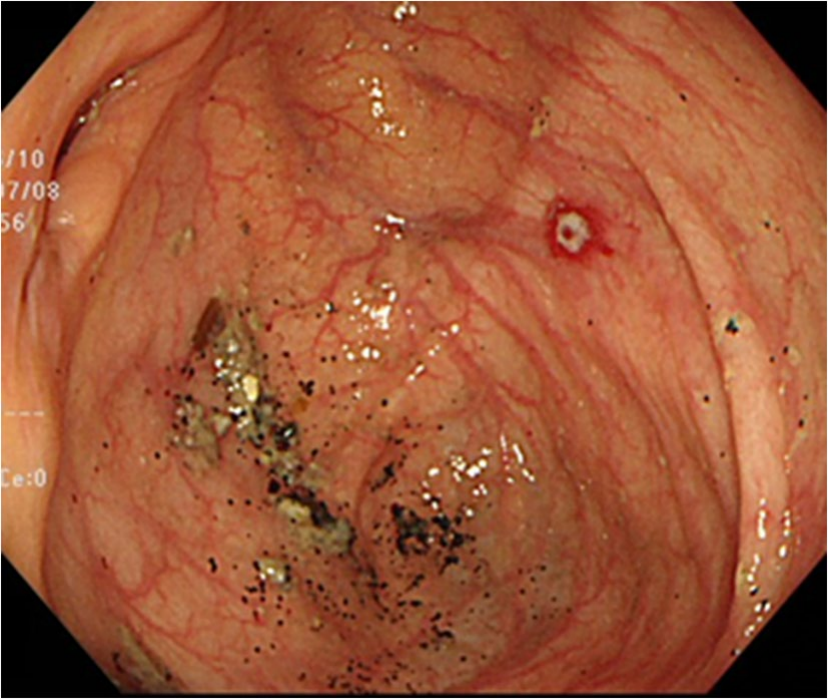
Urgent colonoscopy reveals a protruding small vessel surrounded by a very shallow ulcer in the cecum opposite Bauhin’s valve

**Fig. 2:**
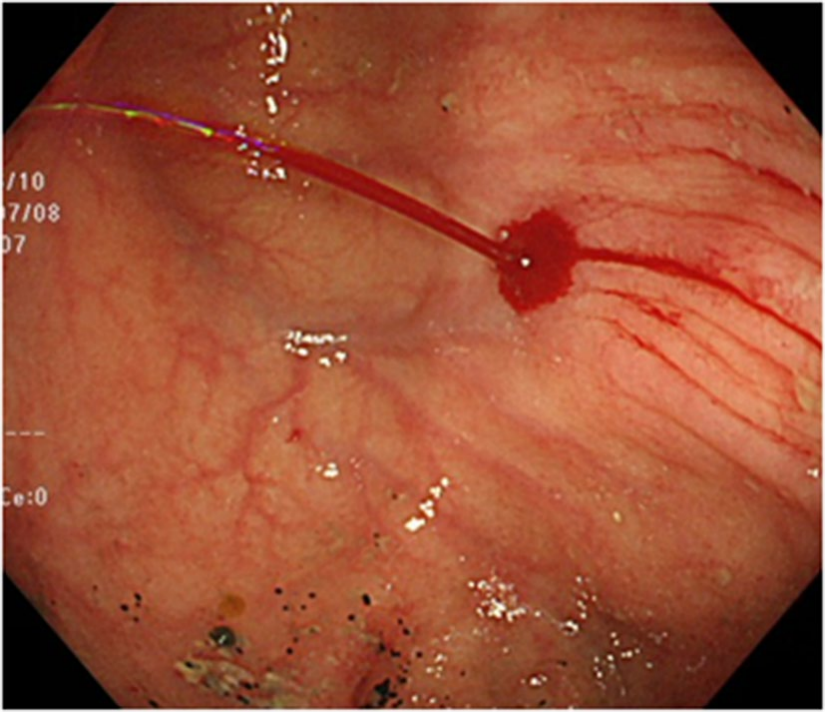
When the reddish point was stimulated by water jet, a spurting bleed occurred

**Fig. 3:**
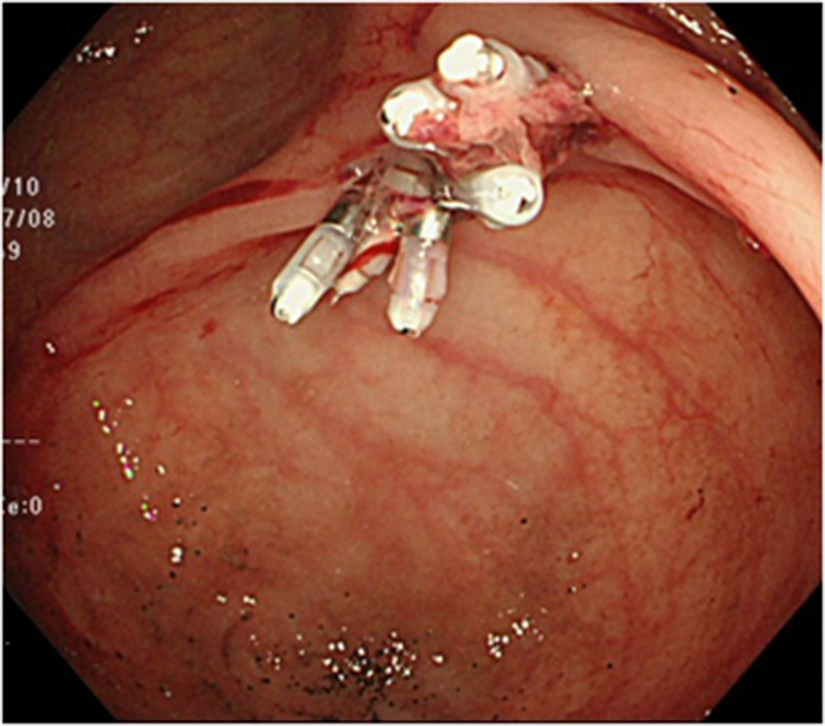
After treatment with endoscopic clipping, no further bleeding is seen from the cecal lesion

Computed tomography revealed no abnormalities in the colon, with no apparent source of gastrointestinal bleeding, vessel malformations or aneurysms. Esophagogastroduodenoscopy showed no hemorrhagic lesion. Urgent colonoscopy revealed a small, protruding vessel surrounded by a very shallow ulcer in the cecum opposite Bauhin’s valve (Fig. 1). When this reddish point was stimulated by water jet from the endoscope, a spurting bleed resulted (Fig. 2). We identified the source of bleeding as the cecum at this point, and suspected Dieulafoy’s ulcer based on the form of the lesion. Treatment was performed by endoscopic clipping (Fig. 3). Follow-up colonoscopy the next day confirmed no bleeding at the cecal lesion. The patient has since been discharged from our hospital without experiencing any further bleeding.

## Discussion

Dieulafoy’s ulcer was first reported as an upper gastric body bleeding lesion by George Dieulafoy, a French surgeon who detailed the anatomy of the malformation in 1898 [4]. Dieulafoy’s ulcer is a very small, superficial ulcerative lesion without swelling and induration in the surrounding mucosa. The ulcer can cause massive bleeding by breaking a relatively thick submucosal artery.

This relatively rare disease comprises 0.3–6.8% of upper gastrointestinal bleeding, most commonly located in the stomach, although any part of the gastrointestinal tract can be affected [5]. Almost two-thirds of lesions are located in the stomach, particularly in the lesser curvature of the gastric body within 6–10 cm of the esophagogastric junction. The remaining one-third of lesions are located in the duodenum and esophagus, followed by the colon (2%) [3, 6]. Colonic lesions are mostly in the rectum (42%), followed by the ascending colon (17%) and cecum (17%) [7, 8]. Dieulafoy’s ulcer often affects the elderly and men, occurring at a median age of 54 years with no familial predisposition [9]. The etiology is considered to involve congenital factors such as vascular malformation, but anticoagulants and antiplatelet drugs and atherosclerotic diseases such as hypertension, diabetes, liver cirrhosis, and chronic kidney disease have been reported as factors potentially contributing to formation of Dieulafoy’s lesions [10–14]. In our case, hypertension and use of antiplatelet drugs were present. To date, the exact mechanisms by which Dieulafoy’s ulcer initiates bleeding are not fully understood. Endoscopy, angiography, scintigraphy and surgery are useful for the diagnosis of Dieulafoy’s ulcer, but correct identification of a bleeding point can be very difficult. Endoscopy is a useful method for diagnosis and treatment and could reduce mortality rate from 80 to 8.6% in patients with hemorrhage due to Dieulafoy’s ulcer [3]. Initial endoscopy is reportedly effective in detecting 49–73% of lesions [5]. However, many cases need multiple endoscopies for diagnosis, and diagnosis from the initial endoscopy may be unlikely. Because the lesion site has poor endoscopic findings other than the bleeding point, a lesion may be missed even if endoscopy is performed without bleeding, due to the nature of the intermittent hemorrhage. Moreover, endoscopic diagnosis of a colonic Dieulafoy’s lesion is often difficult because of the limitations to the view caused by massive bleeding and poor bowel preparation such as blood and stool residue.

No consensus has been reached regarding the treatment of colonic Dieulafoy’s ulcer and no randomized controlled studies have compared different endoscopic therapies. Yoshikumi et al. [15] reported 30 cases of rectal Dieulafoy’s ulcer. Among those cases, 21 patients were treated with endoscopic therapy only, 2 patients were treated with angioembolization and 7 patients were treated surgically. Rebleeding occurred in 5 cases, all of which were treated with endoscopic therapy after rebleeding. With the exception of 1 case, rebleeding tended to occur several days after treatment. However, in that case, rebleeding occurred 5 h after angiographic embolization but it was unclear whether complete hemostasis had been obtained by embolization. Recurrent bleeding was not reported after surgical treatment. Diagnosis is currently made endoscopically in almost all cases, and once the lesion is properly identified, successful hemostasis can be achieved by endoscopic treatment in more than 90% of patients. We, thus, consider endoscopic treatment as the current treatment of choice. Endoscopic treatment is performed by injection of sclerosants such as hypertonic saline epinephrine, pure ethanol, hypertonic glucose, cyanoacrylate and thermic mechanisms such as argon plasma coagulation and heater probe coagulation, mechanical hemostasis with endoscopic clips or band ligation, or even a combination of different hemostatic methods [16–19]. Methods of mechanical hemostasis such as endoscopic clips or band ligation are reportedly better than injection of sclerosants in terms of the number of treatments and the effects of rebleeding suppression [20–22]. Recently, electrical hemostatic forceps are often used for the treatment of GI bleeding and many articles have been reported about the usefulness of hemostatic forceps [23–26]. With colonic lesions, clinicians must beware of the greater risk of perforation, especially rebleeding after thermocoagulation, because of the thinness of the colonic wall compared with the stomach. We, thus, considered that mechanical hemostasis to minimize tissue injury, such as endoscopic clips and band ligation, may offer a better choice for the management of bleeding from colonic Dieulafoy’s ulcer. Angiography is a useful therapy when endoscopy fails. However, the disadvantage of an angiographic approach is the lack of intraoperative direct view and the difficulty of determining the embolization site when bleeding has stopped [16]. Surgery is also a useful therapy when endoscopy and angiography fail, but today these surgical approaches have been replaced by endoscopic and angiographic techniques in most cases.

As mentioned above, we consider mechanic hemostasis with clips as the first-line endoscopic treatment for colonic Dieulafoy’s ulcer because of the simplicity, ready availability, relatively low cost and high utility. Endoscopic band ligation has been reported to achieve complete control of bleeding from Dieulafoy’s ulcer without recurrence [15, 27–29]. In addition, endoscopy can facilitate exact embolization during angiography and help surgeons locate bleeding sites during surgery. Endoscopy is, therefore, useful in the treatment of colonic Dieulafoy’s ulcer.

Although Dieulafoy’s ulcer is a rare disease, we should consider the disorder when no source of gastrointestinal bleeding can be identified, because this pathology causes lower gastrointestinal bleeding and hemorrhagic shock. We have reported a case of lower gastrointestinal bleeding from a cecal Dieulafoy’s ulcer that was successfully treated with endoscopic clipping. Our case suggested that cecal Dieulafoy’s ulcer may have been caused by aspirin.
